# Endoscopic ultrasound-guided fine-needle biopsy of pancreatic hepatoid adenocarcinoma

**DOI:** 10.1055/a-2623-6139

**Published:** 2025-07-02

**Authors:** Yuping Zhang, Honglong Wei, Guihui Zhang, Haiyan Dong

**Affiliations:** 1 66310 Department of Gastroenterology, The First Affiliated Hospital of Shandong First Medical University and Shandong Provincial Qianfoshan Hospital, Jinan, China; 2 66310 Department of General Surgery, The First Affiliated Hospital of Shandong First Medical University and Shandong Provincial Qianfoshan Hospital, Jinan, China; 3 66310 Department of Pathology, The First Affiliated Hospital of Shandong First Medical University and Shandong Provincial Qianfoshan Hospital, Jinan, China

**Fig. 1:**
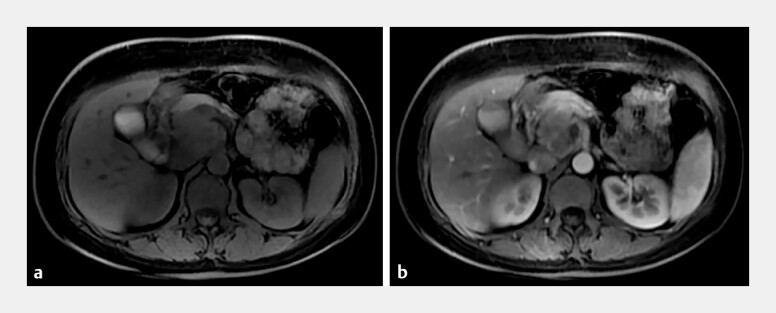
**a** Contrast-enhanced magnetic resonance imaging revealed an abnormal signal focus in the head of the pancreas, appearing heterogeneously isointense to slightly hyperintense on T2-weighted imaging. **b** The lesion demonstrated heterogeneous moderate enhancement during the arterial phase on contrast-enhanced imaging.

We report the case of a 46-year-old woman who presented with a 4-day history of abdominal pain. Contrast-enhanced magnetic resonance imaging revealed a 37 × 41-mm lesion in the head of the pancreas, which appeared slightly hypointense on T1-weighted imaging and heterogeneously isointense to slightly hyperintense on T2-weighted imaging ( Fig. 1 **a** ). The lesion demonstrated heterogeneous enhancement on contrast-enhanced scans, with moderate enhancement observed during the arterial phase ( Fig. 1 **b** ). The female tumor marker panel revealed a markedly elevated alpha-fetoprotein (AFP) level of 792.1 ng/mL (reference range: 0–7 ng/mL).

**Fig. 2:**
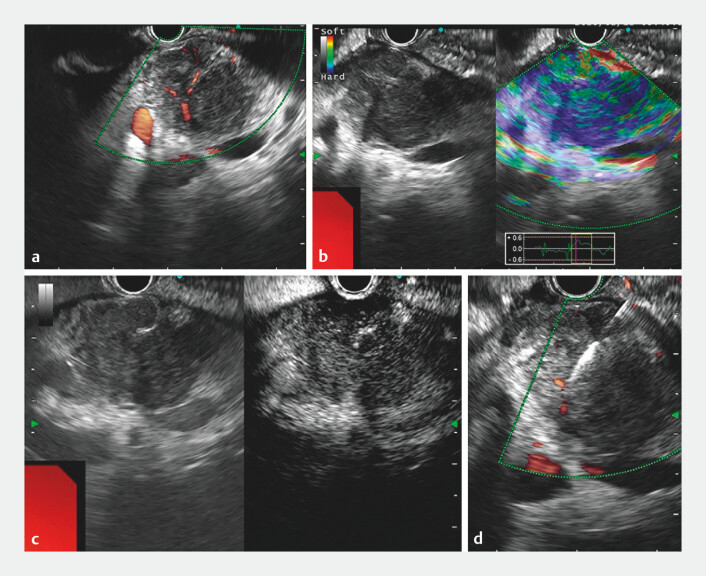
**a–b** Endoscopic ultrasound (EUS) revealed an irregular hypoechoic mass in the head of the pancreas, with abundant intralesional blood flow signals ( **a** ) and a firm texture on elastography ( **b** ). **c** Contrast-enhanced harmonic EUS demonstrated hyperenhancement during the arterial phase. **d** EUS-guided fine-needle biopsy of the lesion was performed.

To achieve a definitive diagnosis, endoscopic ultrasound-guided fine-needle biopsy (EUS-FNB) was performed ( Video 1 ). EUS identified an irregular, hypoechoic mass in the head of the pancreas, with abundant intralesional blood flow on Doppler imaging ( Fig. 2 **a** ) and a firm consistency on elastography ( Fig. 2 **b** ). Contrast-enhanced harmonic EUS demonstrated hyperenhancement during the arterial phase ( Fig. 2 **c** ) and hypoenhancement in the venous phase. The biliary and pancreatic ducts were not dilated. To make a pathological diagnosis, EUS-FNB of the lesion was performed using a 22-gauge EZShot3Plus FNB needle without side holes (Olympus Corp., Tokyo, Japan) ( Fig. 2 **d** ).

A definitive diagnosis of hepatoid adenocarcinoma of the pancreas was established via endoscopic ultrasound-guided fine-needle biopsy (EUS-FNB) in a 46-year-old woman.Video 1

**Fig. 3:**
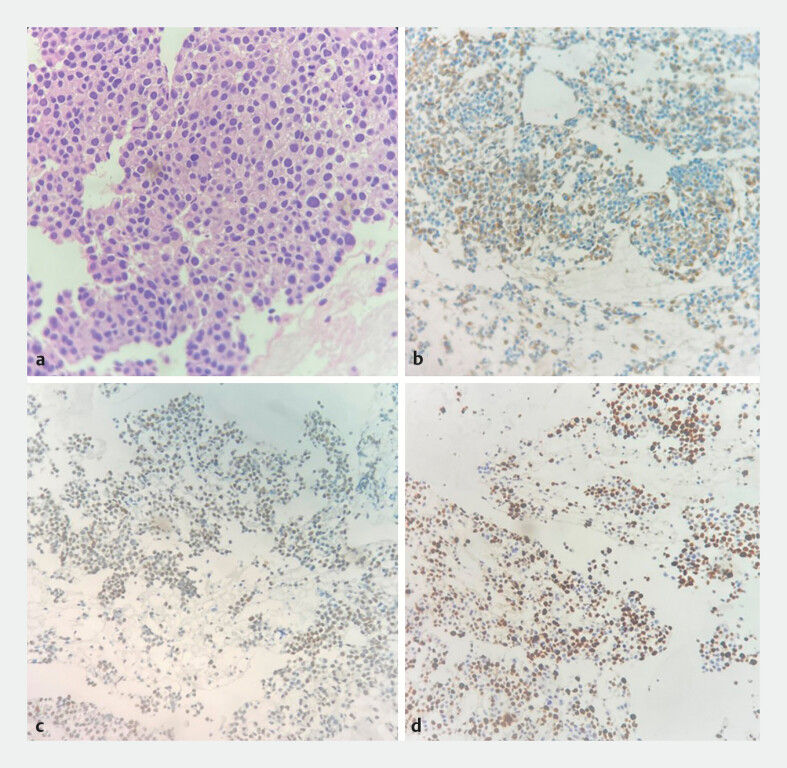
**a** Histopathological examination revealed that the tumor was composed of solid, sheet-like clusters of cells with hepatocyte-like morphology. The cells were oval-to-polygonal, with eosinophilic cytoplasm, well-defined borders, large and irregular nuclei, uneven chromatin distribution, and readily identifiable mitotic figures. **b–c** Immunohistochemical staining demonstrated positivity for SALL4 ( **b** ) and AFP ( **c** ). **d** The Ki-67 proliferation index was approximately 50%.

Histopathological examination revealed a tumor composed of solid, sheet-like clusters of cells with hepatocyte-like morphology ( Fig. 3 **a** ). Immunohistochemical analysis demonstrated co-expression of SALL4 and AFP ( Fig. 3 **b, c** ), strong Villin positivity (4+), and absence of Glypican-3 and Hepatocyte marker staining, supporting the diagnosis of hepatoid adenocarcinoma. The Ki-67 proliferation index was 50% ( Fig. 3 **d** ).

Subsequent positron emission tomography–computed tomography showed no evidence of metastatic disease outside the pancreas. Based on these findings, the patient was definitively diagnosed with pancreatic hepatoid adenocarcinoma (PHA) and referred to the oncology department for further management.

PHA is a rare malignant tumor of the pancreas. To our knowledge, this is the first reported case in which EUS-FNB was employed for the preoperative diagnosis of PHA. This case highlights the critical role of EUS-FNB in the diagnostic evaluation of PHA.

Endoscopy_UCTN_Code_CCL_1AZ_2A

